# All-*Trans*-Retinoic Acid Combined With Valproic Acid Can Promote Differentiation in Myeloid Leukemia Cells by an Autophagy Dependent Mechanism

**DOI:** 10.3389/fonc.2022.848517

**Published:** 2022-02-24

**Authors:** Dalyia N. Benjamin, Tracey R. O’Donovan, Kristian B. Laursen, Nina Orfali, Mary R. Cahill, Nigel P. Mongan, Lorraine J. Gudas, Sharon L. McKenna

**Affiliations:** ^1^ Cancer Research, University College Cork, Cork, Ireland; ^2^ Department of Haematology, Tallaght University Hospital, Dublin, Ireland; ^3^ Department of Pharmacology, Weill Cornell Medical College of Cornell University, New York, NY, United States; ^4^ Department of Haematology, St James’s Hospital, Dublin, Ireland; ^5^ Department of Haematology, Cork University Hospital, Cork, Ireland; ^6^ Faculty of Medicine and Health Science, Biodiscovery Institute, University of Nottingham, Nottingham, United Kingdom

**Keywords:** APL, AML, ATRA, valproic acid, autophagy, differentiation, TFEB, ATG7

## Abstract

Acute myeloid leukemia (AML) is an aggressive blood cancer with an overall survival of 30%. One form of AML, acute promyelocytic leukemia (APL) has become more than 90% curable with differentiation therapy, consisting of all-*trans*-retinoic acid (ATRA) and arsenic trioxide (ATO). Application of differentiation therapy to other AML subtypes would be a major treatment advance. Recent studies have indicated that autophagy plays a key role in the differentiation of ATRA-responsive APL cells. In this study, we have investigated whether differentiation could be enhanced in ATRA resistant cells by promoting autophagy induction with valproic acid (VPA). ATRA sensitive (NB4) and resistant leukemia cells (NB4R and THP-1) were co-treated with ATRA and valproic acid, followed by assessment of autophagy and differentiation. The combination of VPA and ATRA induced autophagic flux and promoted differentiation in ATRA-sensitive and -resistant cell lines. shRNA knockdown of ATG7 and TFEB autophagy regulators impaired both autophagy and differentiation, demonstrating the importance of autophagy in the combination treatment. These data suggest that ATRA combined with valproic acid can promote differentiation in myeloid leukemia cells by mechanism involving autophagy.

## Introduction

Acute myeloid leukemia (AML) is a group of haematopoietic disorders characterised by a failure of differentiation in myeloid progenitor cells. The clonal expansion of immature cells impairs critical aspects of haematopoiesis and requires urgent intervention. Prognosis depends on multiple factors including age, patient co-morbidity and the presence of cytogenetic and genetic abnormalities in leukemic clones (1). The outcome for adults with AML remains poor with a 5-year survival rate of ~40% for younger patients (18–60 years) and until recently was as low as ~10% for patients above the age of 60 years (2, 3). While therapies have advanced in the last 5 years (4), there remains an urgent need for better, well tolerated, outpatient-based management strategies in older adults. One particular subgroup of AML, acute promyelocytic leukemia (APL), which represents about 5-10% of cases, has undergone a radical improvement in treatment outcomes in the last 30 years with the advent of differentiation therapy. Current chemotherapy-free treatment regimens for low to intermediate-risk APL, based on all-*trans*-retinoic acid (ATRA) and arsenic trioxide (ATO), achieve complete remission rates exceeding 90% and overall survival of 85-99% (5).

Most APL cells have a specific cytogenetic abnormality involving a translocation between chromosomes 15 and 17 t (15,17), leading to the formation of an abnormal fusion gene *PML/RARα*. The PML-RARα oncoprotein binds to retinoic acid response elements (RAREs) on retinoid target gene promoters and has a high affinity for co-repressor histone deacetylases (HDACs) (6, 7). This leads to repressive epigenetic changes that inhibit the transcription of RARα target genes required for differentiation (8). In addition, PML-RARα disrupts the organisation of nuclear PML bodies (9). When ATRA binds to PML/RARα, co-repressors are released and co-activators including histone acetyltransferases (HATS) are recruited to decompress chromatin and initiate transcription (10, 11). ATRA also influences the stability of the PML-RARα oncoprotein by promoting its degradation through co-operating proteolytic mechanisms [reviewed in (12)]. The introduction of arsenic trioxide (ATO) into the protocol can eliminate the leukemic stem cell, generating a lasting cure (13).

The evolution of APL therapy away from chemotherapy has been a remarkable achievement and the application of this approach to other forms of AML would be hugely advantageous, particularly for older patients, who are often ineligible for intensive chemotherapy due to co-morbidities. However, despite an increased understanding of the molecular abnormalities in other AML subtypes, no targeted agent has been developed that can overcome the repression of differentiation to the extent achieved with ATRA in APL. One approach has been to globally release the repression of gene expression using inhibitors of epigenetic regulators. Several epigenetic drugs have either been approved for AML or are in clinical development [reviewed in (14)]. It is currently unclear how much of their anti-leukemic activity is related to effects on differentiation – as direct induction of apoptosis and cell cycle arrest are also reported activities of these agents (15).

Autophagy has now been identified as a key component of differentiation in myeloid leukemia cells. Canonical autophagy is a highly conserved catabolic process in which cells self-digest organelles and other macromolecular complexes by forming a double-membraned vesicle around sequestered cytoplasmic material, termed the autophagosome. Fusion of the autophagosome with a lysosome generates an autolysosome, which facilitates the degradation and recycling of macromolecules back to the cytoplasm for anabolic activities. At least two forms of alternative autophagy have been characterised, mainly through autophagy gene knockouts in mouse embryonic fibroblasts. These include (i) BECN1-independent autophagy and (ii) ATG5- and ATG7-independent autophagy. In conventional autophagy, autophagosome biogenesis is initiated at various intra-cellular membranes such as the ER and mitochondria, whereas in alternative autophagy, the Golgi is the primary membrane source for autophagosomes. Both forms of alternative/non-canonical autophagy are inhibited by the Golgi inhibitor and anti-viral agent, brefeldin A (BFA) (16–18).

Autophagy is important for various aspects of haematopoiesis including the cellular remodelling involved in differentiation (19). A compelling role for autophagy in the development of AML has come from knockout studies of key autophagy genes. Loss of either ATG7 or ATG5 in haematopoietic stem and progenitor cells (HSPC) leads to a lethal pre-leukemic phenotype in mice (20, 21). In addition, heterozygous loss of ATG5 in a MLL-ENL murine model of AML led to a more aggressive leukemic phenotype (21). These studies indicate that effective autophagy will protect against leukemic transformation and is consistent with other studies suggesting compromised autophagy in AML (22, 23).

We and others have demonstrated the importance of autophagy in ATRA-induced differentiation (24). We subsequently conducted RNAseq analysis following ATRA treatment of APL cells and found that 84 genes implicated in autophagy were differentially expressed. One of these genes was *TFEB*, a master regulator of autophagy and lysosome biogenesis. shRNA‐mediated depletion of TFEB impacted both autophagy and expression of differentiation associated genes. Interestingly, inhibition of alternative autophagy with BFA also impeded ATRA-induced autophagy and differentiation suggesting more than one autophagy pathway is likely to be involved in differentiation (25).

In the current paper we investigate the possibility of enhancing differentiation through the promotion of autophagy. To this end, we sought a clinically approved agent with (i) a reasonable toxicity profile and (ii) reported activity in the induction of both autophagy and differentiation. Valproic acid (VPA), is a HDAC inhibitor with activity against Type I and IIa histone deacetylases (15). It has been used safely for decades to treat epilepsy and bipolar disorder and is well tolerated by cancer and leukemia patients (26, 27). VPA has previously been reported to have anti-leukemic effects and to promote differentiation in the presence of ATRA (28, 29). It has also been demonstrated to induce autophagy (30–33). It is not known whether these activities are directly or indirectly linked. It is also not known whether autophagy is important for the activity of the combination treatment, as the HDAC inhibitory activity of VPA may induce several co-operating pathways.

We have therefore examined VPA as a co-agent with ATRA, for the treatment of sensitive and resistant APL (NB4 and NB4R cells) and non-APL AML leukemia cells (THP-1 cells), which are differentiation resistant. We have evaluated both differentiation and autophagy induced by the compounds alone and in combination. We have demonstrated the importance of autophagy in this drug combination through shRNA knockdown of the ATG7 and TFEB autophagy regulators in NB4 cells. In addition, BFA treatment inhibits VPA and ATRA induced autophagy, indicating the possibility of alternative autophagy pathways playing a role in the differentiation induced. These data suggest that the induction of autophagy is a key component of an effective differentiation-inducing regimen in myeloid leukemia cells.

## Materials And Methods

### Cell Culture and Drug Treatments

NB4 human APL cells were a gift from Dr. David Scheinberg (Memorial Sloan Kettering Institute, New York, NY, USA). The ATRA-resistant NB4R cell line was a gift from Prof. Pier Paolo-Pandolfi (Beth Israel Deaconess Cancer Centre, Boston, MA, USA). Cells were cultured at 37°C, 5% CO_2_ in HyClone RPMI 1640 medium (GE Healthcare Life Sciences SH30027), supplemented with 10% foetal calf serum (Sigma-Aldrich, F7524) and 1% penicillin/streptomycin (Invitrogen, 10378-016). THP-1 cells (monocytic-lineage) were from the American Type Culture Collection (ATCC). Cells were cultured in RPMI 1640 (1x) medium (Gibco by Life technologies, 21870-076), supplemented with 10% foetal calf serum and 1% penicillin/streptomycin. Cells were seeded at 5x10^4^ cells/ml prior to treatment. ATRA (Sigma-Aldrich, R2625) was used to induce differentiation at 1 μM, diluted from a 1 mM stock in 100% ethanol (EtOH). Control populations were treated with 0.1% v/v EtOH. Valproic acid (Sigma-Aldrich, P4543) was used at 1 mM concentration, diluted from a 500mM stock in H_2_0.

### RNA Extraction, Reverse Transcription, Quantitative PCR (RT-qPCR)

Total cellular RNA was harvested using TriZol (Invitrogen, 15596-018), according to the manufacturer’s protocol. Total RNA (1μg) was reverse transcribed using qScript (Quanta Biosciences, 95047) as per product protocol at a final reaction volume of 20μL and the resulting cDNA was diluted 1:10 in H_2_O. Subsequent qRT-PCR reactions were carried out using 2μL of template together with 1x SYBR Green Supermix (Quanta Biosciences, 84091), forward and reverse primers at 0.25uM and 2.5μL H_2_O in a final reaction volume of 15μL. Reactions were run on a Bio-Rad MyiQ™ Single Colour Real-time PCR detection system with each cycle including a 94°C x 20sec denaturation step, 60°C x 20sec annealing step and a 72°C x 30sec extension step. Primer pairs are listed in **Table 1**. The transcript levels in biological replicates (n=6) were normalized to *HPRT* transcript levels and relative differences were calculated using the Pfaffl method (34). Graphical displays and measurements of statistical significance were performed on GraphPad Prism software.

**Table 1:** List of Primer pairs used.

	Gene Name	Forward Primer	Reverse Primer
**Differentiation associated**	CD11β	ATGGAGTTCAATCCCAGGGAAG	GAGTCCAGAGCCAGGTCATAAG
	*ID2*	CACTGTGTGGCTGAATAAGCGGTGT	GTAAGAGAACACCCTGGGAAGATG
	*GCSFR*	ATCCTGGACTGCGTGCCCAAG	AGCATGGGGGGCTCCAGTTTCA
	*CEBP*	ACAATCCCCTGCAGTACCAAG	ACTGCCTTCTTGCCCTTGTG
**Autophagy associated**	*CTSD*	TGCTCAAGAACTACATGGACGC	CGAAGACGACTGTGAAGCACT
	*GABARAP*	GGGCGAGAAGATCCGAAAGA	TCCAGGTCTCCTATCCGAGC
	*TFEB*	AAGCGAGAGCTCACAGATGC	TGAGGATGGTGCCCTTGTTC
	*ATG16L1*	TTGCAAGCCGAATCTGGACTGT	GGTCGTGACTTCCTGAGACAAT
**Control**	*HPRT*	TGCTCGAGATGTGATGAAGG	TCCCCTGTTGACTGGTCATT

### Morphology

Cells were cytospun onto glass slides and stained with Rapi-Diff (Braidwood Laboratories, 22007, 22008, 22009) according to product guidelines. Granulocytic differentiation was assessed by light microscopy using an Olympus DP70 digital microscope (Mason Technology, Ireland). Cells with lobular/sub-divided nuclei were scored as differentiated.

### Western Blotting

Cells were lysed in modified RIPA buffer (50 mM TrisHCl - pH 7.4, 150 mM NaCl, 0.25% sodium deoxycholate, 1% Igepal, 1 mM EDTA, 1x Pefabloc, 1x Protease inhibitor cocktail, 1 mM Na_3_VO_4_, 1 mM NaF). Protein samples were separated on NuPAGE 4-12%, Bis-Tris gels (Invitrogen, NP0322) and electrophoretically transferred onto PVDF membranes (Invitrogen, IB401001). Membranes were incubated with anti-LC3 (MBL, PD014) antibody, diluted 1/500 in 5% milk overnight, at 4°C and with ß-actin (loading control) (Sigma-Aldrich, A5441) for 1 hour at room temperature. Proteins were visualized using relevant IR-DYE secondary antibodies and quantified on the Odyssey IR imaging system (Li-Cor, Cambridge, UK). All bands were quantified, normalised to ß-actin and presented as integrated intensities, with all bands normalised to untreated control cells. For all western blots, integrated intensities shown are representative of three independent experiments.

### Flow Cytometry

Live cells were incubated for 30 min with PE-conjugated anti-CD11b antibody (eBioscience, 12-0118) in 1% albumin/phosphate buffered saline (PBS), and washed with PBS prior to analysis. Fluorescence was detected using a BD-LSRII flow cytometer (BD Biosciences, Oxford, UK). Data analysis and histogram overlays were performed on FlowJo software.

*ATG7 and TFEB knockdown cells.* ATG7 and TFEB knockdown cell lines were previously generated using shRNA vectors targeting ATG7 and TFEB and non-gene targeting scrambled controls (Scr). Gene expression levels and effect on downstream targets is detailed in previous publications (24, 25).

### Statistical Analysis

Statistical analysis was carried out using GraphPad Prism 5 software. Means were compared using independent student t-tests (unpaired). The p-value was considered statistically significant when it was *p < 0.05, **p < 0.005, ***p < 0.0005.

## Results

### VPA Promotes ATRA-Mediated Differentiation in ATRA Sensitive and Resistant Cell Lines

**Figure 1:**
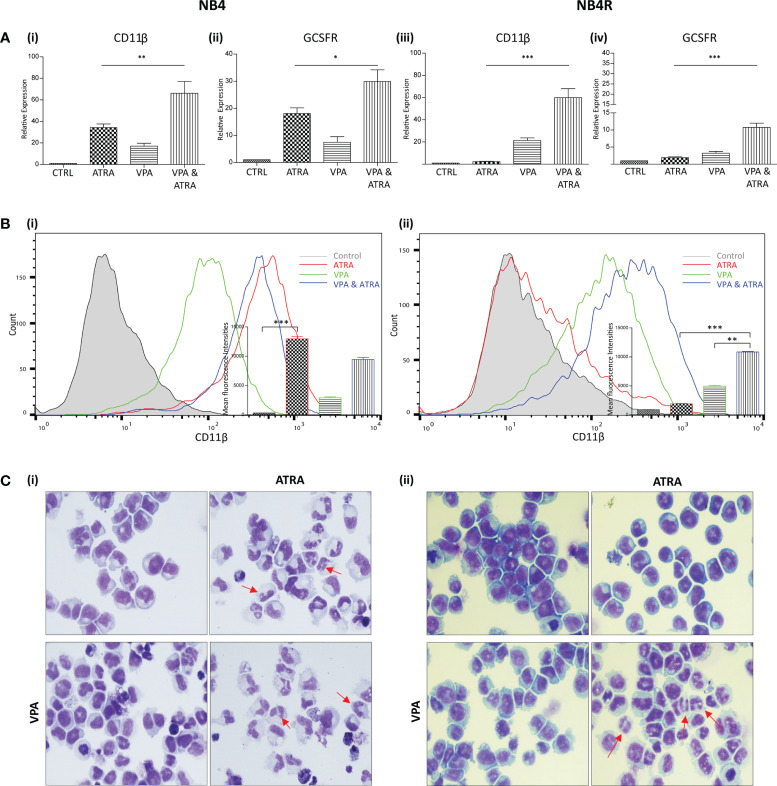
Valproic acid (VPA) promotes all-*trans*-retinoic acid (ATRA)-mediated myeloid differentiation in ATRA sensitive and ATRA resistant, acute promyelocytic leukemia (APL) cell lines (NB4 and NB4R). NB4 and NB4R cell lines were treated with either ATRA (1 μM) or VPA (1mM) alone or in combination for 72 hours. Induction of differentiation was assessed by measuring **(A)** expression levels of known differentiation genes CD11β **(i, iii)** and GCSFR **(ii, iv)** by RT-qPCR. Raw Ct values were normalised to a housekeeping gene and data are shown as n-fold induction compared to untreated controls (n = 3). **(B)** Expression of surface CD11β (eBioscience antibody) by flow cytometry was assessed in NB4 **(i)** and NB4R **(ii)** cells. Colours are as follows: control untreated (grey histogram), ATRA (red overlay), VPA (green overlay) and VPA&ATRA treated cells (blue overlay). A single representative histogram is shown, with mean fluorescence intensity ± SEM presented in the inset graph (n = 4). ***p < 0.0005, **p < 0.005, *p < 0.05. **(C)** Morphological features of granulocytic differentiation were assessed by light microscopy (400x) in NB4 **(i)** and NB4R **(ii)** cells. Red arrows indicate cells with lobular/sub-divided nuclei, a feature of granulocytic differentiation.

We investigated whether valproic acid (VPA) alone and in combination with all-*trans*-retinoic acid (ATRA) could induce differentiation in ATRA sensitive and resistant acute promyelocytic leukemia (APL) cell lines (NB4, NB4R) (**Figure 1** and **Supplementary Figure 1**) and in a non-APL, differentiation resistant myeloid leukemia cell line (THP-1) (**Supplementary Figures 2A, B**). Cells were treated with VPA (1 mM) or ATRA (1 µM) alone or in combination for 72 hours. For clarity, the combination treatment is referred to in the figures as “VPA & ATRA”. Expression levels of four known markers of myeloid differentiation were evaluated by RT-qPCR. Data for *CD11β* and granulocyte colony-stimulating factor receptor (*GCSFR*) are shown in **Figure 1A**, with additional differentiation markers, *CEPBε* and *ID2*, shown in **Supplementary Figure 1**. In NB4 cells, expression of (i) *CD11β* and (ii) *GCSFR* was significantly increased in the combination treatment compared to treatment with either agent alone (p = 0.0077 and p = 0.0197 respectively). In the ATRA-resistant NB4R cells, ATRA alone had no significant effect on transcript levels, but the combination of VPA and ATRA induced a significant increase in both differentiation markers [**Figure 1A** **(iii)** and **(iv)**] (p < 0.0001). Similar to the NB4R cells, THP-1 cells displayed little or no *CD11β* or *GCSFR* expression in response to ATRA alone, but we observed a significant increase in the combination treated cells (**Supplementary Figure 2A**) (p < 0.0001 and p = 0.0071 respectively).

Surface expression of CD11β protein was also examined by flow cytometry (**Figure 1B**). As shown previously (24), surface CD11β expression is significantly elevated in response to ATRA alone (red histogram overlay) in NB4 cells (p < 0.0001) and this was not further enhanced by the addition of VPA (blue overlay) [**Figure 1B** **(i)**]. The NB4R cells however, display a significant increase in surface CD11β in combination treated cells (blue overlay) when compared to either ATRA (red overlay) (p < 0.0001) or VPA alone (green overlay) (p = 0.0011) [**Figure 1B** **(ii)**]. In THP-1 cells, surface CD11β is also significantly enhanced in the combination treated cells compared to ATRA (p = 0.0014) or VPA alone (p = 0.004) (**Supplementary Figure 2B**).

Morphological assessment shows granulocytic differentiation in ATRA-treated and combination treated NB4 cells (denoted with red arrows) [**Figure 1C** **(i)**]. In the ATRA resistant NB4R cells, differentiation is only evident with the combination of VPA and ATRA [**Figure 1C** **(ii**), lower right panel]. In THP1 cells, morphological features of differentiation were also only observed with the combination treatment (**Supplementary Figure 2C**).

### VPA Promotes ATRA-Induced Autophagy in ATRA Sensitive and Resistant Cell Lines

**Figure 2:**
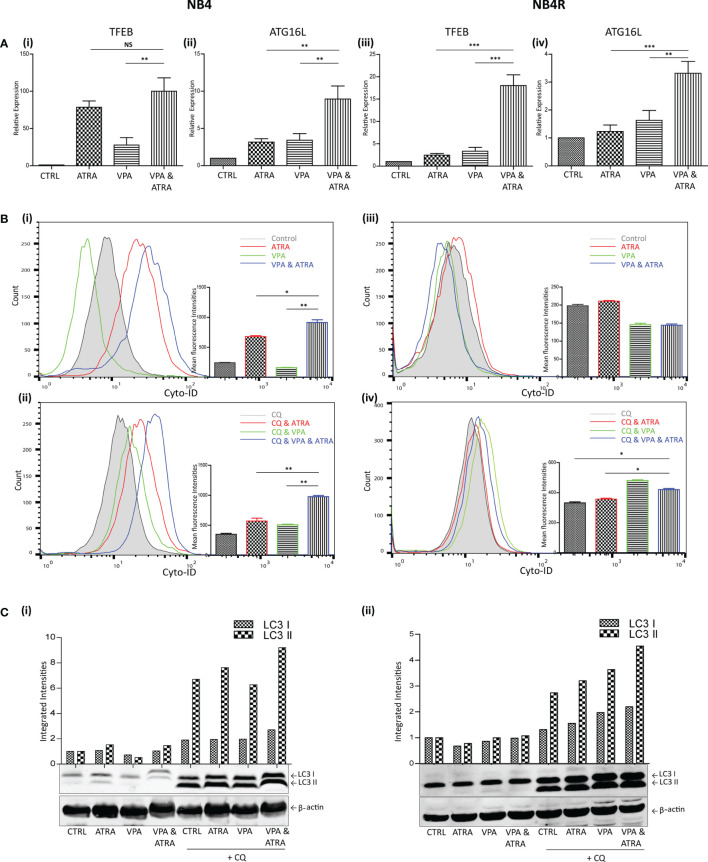
Valproic acid (VPA) promotes all-*trans*-retinoic acid (ATRA)-induced autophagy in ATRA sensitive and ATRA resistant, acute promyelocytic leukemia (APL) cell lines (NB4 and NB4R). NB4 and NB4R cell lines were treated with either ATRA (1 μM) or VPA (1mM) alone or a combination of both for 72 hours. To assess autophagy flux, chloroquine (CQ) (10 µM) was added 2 hours prior to the addition of ATRA, VPA or combination treatment. Autophagy induction was assessed by measuring **(A)** mRNA expression of autophagy regulators, TFEB **(i, iii)** and ATG16L **(ii, iv)** by RT-qPCR. Raw Ct values were normalised to a housekeeping gene and data are shown as n-fold induction compared to untreated controls (n = 3). **(B)** Cyto ID was used to assess autophagosome formation in NB4 **(i)** and NB4R **(iii)** cells in response to ATRA (red overlay), VPA (green overlay) and VPA&ATRA (blue overlay) relative to untreated controls (grey histogram). Flux was analysed in NB4 **(ii)** and NB4R **(iv)** cells with the addition of CQ to all treatments, which are then assessed relative to CQ alone-control cells (grey histogram). Data from three independent experiments is presented in the inset graph as mean fluorescence intensities ± SEM ***p < 0.0005, **p < 0.005, *p < 0.05. **(C)** Autophagic flux was assessed by western blot analysis of LC3II levels in NB4 **(i)** and NB4R **(ii)** cells, following treatment with ATRA, VPA or a combination of both, in the absence (lanes 1-4) and presence of chloroquine (CQ) (lanes 5-8). An increase in LC3II in cells treated with VPA, ATRA & CQ (lane 8), beyond that induced with chloroquine alone (lane 5) is indicative of flux. All bands were quantified using the Odyssey Infrared Imaging System (Li-COR), normalised to ß-actin and presented as integrated intensities, with all bands normalised to untreated control cells (lane 1). For all western blots, integrated intensities are representative of three independent experiments (n = 3). NS, not significant.

We and others have previously shown that ATRA induces autophagy in the APL NB4 cell line (24). Here, we investigate whether VPA can enhance the autophagy induced by ATRA in NB4 cells and whether it can help to initiate autophagy in the ATRA-resistant NB4R and THP-1 cells. Cells were treated with VPA (1 mM) or ATRA (1 µM) alone or in combination for 72 hours. Induction of autophagy was initially examined by RT-qPCR assessment of key autophagy genes, identified from our previous RNAseq analysis as induced in ATRA sensitive cells (25). Expression of both *TFEB* and *ATG16L* increased in response to ATRA or VPA alone in ATRA-sensitive NB4 cells, with a further significant increase in cells treated with the combination [**Figure 2A** **(i, ii)**] (p = 0.0013, 0.0078 respectively). ATRA alone had no significant effect on *TFEB* and *ATG16L* transcript levels in NB4R cells, but the addition of VPA to ATRA induced a significant increase in expression of both *TFEB* and *ATG16L* [**Figure 2A** **(iii, iv)]** (p < 0.0001, = 0.0001 respectively). Additional autophagy associated genes *GABARAP* and *CTSD* are shown in **Supplementary Figure 3**.

The presence of autophagosomes was then quantified by flow cytometry, using the fluorescent autophagosome marker Cyto-ID (**Figure 2B**). ATRA treatment induced autophagosome accumulation in the NB4 cell line (red overlay), which was significantly increased by the addition of VPA (blue overlay) (p = 0.0208), [**Figure 2B** **(i)**]. VPA alone induced a backward shift (green overlay), indicating that less autophagosomes accumulate in the presence of VPA [**Figure 2B** **(i)**]. This suggests that VPA is either inhibiting autophagy or promoting faster turnover of autophagosomes. To distinguish this, cells were treated with the lysosome inhibitor, chloroquine (CQ), (10 µM) for two hours prior to the addition of VPA, ATRA or VPA with ATRA. CQ raises lysosomal pH, inhibiting autophagosome-lysosome fusion. CQ treatment alone will therefore cause an increase in fluorescence due to a block in the turnover of existing/basal autophagosomes. In cells co-treated with another agent, accumulation of autophagosomes beyond that observed with CQ alone represents new autophagosome production.

In **Figure 2B** **(ii, iv)**, all cells are treated with CQ. In NB4 cells, the addition of CQ caused a shift of the grey histogram to the right [**Figure 2B** **(ii)**], (compared to the grey histogram in [**Figure 2** **B(i)**], indicative of a block in the turnover of basal autophagy. Interestingly, VPA also demonstrated increased autophagosome accumulation in the presence of CQ (green histogram). This indicates that VPA is an inducer of autophagy, and that the backward shift in fluorescence with the VPA treatment noted above [**Figure 2B** **(i)**] is likely to be due to enhanced lysosomal processing of autophagosomes. It is possible that valproic acid may enhance the delivery to lysosomes, or increase the availability of lysosomes for autophagosome turnover. Indeed, the increased expression of TFEB (a master regulator of lysosome biogenesis), in response to VPA (shown in **Figure 2A**), may contribute to this effect.

Further autophagosomes accumulated in NB4 cells in the presence of ATRA and CQ (red overlay) and this was again enhanced by the VPA&ATRA combination [**Figure 2B** **(ii)**, blue overlay] (p = 0.005), demonstrating that autophagic flux is significantly induced by the combination treatment.

In the NB4R cells, autophagy was only evident when CQ was added, with a significant increase seen in the combination of VPA and ATRA [**Figure 2B** **(iv)**, blue overlay], compared to CQ, or ATRA and CQ (p = 0.017 and 0.0229 respectively.)

Induction of autophagy was also confirmed by examining the levels of a known autophagy marker, LC3. LC3I becomes conjugated to phosphatidylethanolamine (PE), to form LC3II, which is incorporated into forming autophagosome membranes. Accumulation of LC3II was assessed by western blot (**Figure 2C**). Both NB4 and NB4R cell lines demonstrated basal autophagy flux with the addition of CQ (lane 5 (i) and (ii)), with an increased accumulation in VPA&ATRA treated cells (lane 8), beyond that observed with CQ or ATRA and CQ (lanes 5 & 6).

In a similar pattern to NB4R cells, THP-1 cells displayed little or no difference in transcript levels of either *TFEB* or *ATG16L* in response to ATRA alone, with a significant increase observed with the addition of VPA to ATRA [**Supplementary Figure 2D (i, ii)**], (p = 0.0131, 0.0127). Evidence of autophagy flux was only obvious when CQ was added [**Supplementary Figure 2E (ii, iii)**], noted in both Cyto-ID and western blot analysis (suggesting rapid autophagosome turnover), with significantly higher levels of autophagosomes in the combination treatment (blue overlay and lane 8), compared to ATRA alone (red overlay and lane 5) (p = 0.0144).

Collectively these data demonstrate that VPA promotes both ATRA-induced myeloid differentiation and autophagy in ATRA sensitive and ATRA resistant, APL and non-APL cell lines.

### shRNA-Mediated Depletion of ATG7 Attenuates VPA and ATRA-Induced Autophagy and Differentiation in APL Cells

**Figure 3:**
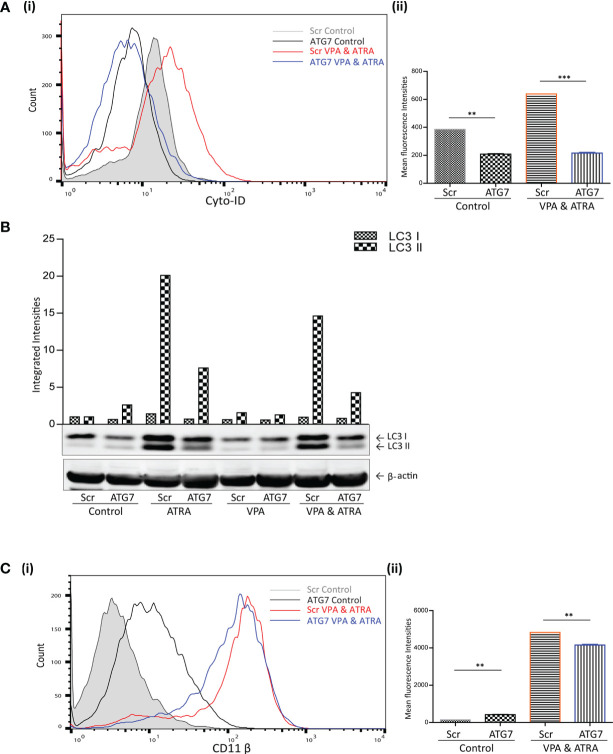
ATG7 knockdown attenuated valproic acid (VPA) & all-*trans*-retinoic acid (ATRA)-induced autophagy and differentiation. ATG7 was knocked down in NB4 cells using lentiviral transduction of target-specific short hairpin (sh)RNA (ATG7). NB4 cells were also transduced with an off-target scrambled shRNA (Scr). Both cell lines were treated with either ATRA (1 μM) or VPA (1mM) alone or a combination of both for 72 hours. **(A) (i)** Cyto-ID was used to assess autophagosome content in control untreated Scr (grey histogram), untreated ATG7 knockdown (black overlay), VPA&ATRA treated Scr (red overlay) and ATG7 knockdown (blue overlay) cell lines. **(ii)** Data from three independent experiments is presented in the graph to the right, as mean fluorescence intensities ± SEM ***p < 0.0005, **p < 0.005. **(B)** Western blot analysis of LC3II levels in Scr and ATG7 knockdown cells, following treatment with ATRA, VPA or a combination of both. All bands were quantified using the Odyssey Infrared Imaging System (Li-COR), normalised to ß-actin and presented as integrated intensities, with all bands normalised to Scr untreated control cells (lane 1). For all western blots, integrated intensities are representative of three independent experiments (n = 3). **(C) (i)** The induction of differentiation was assessed by measuring expression of surface CD11β by flow cytometry in untreated Scr (grey histogram), untreated ATG7 KD (black overlay), and VPA&ATRA treated Scr (red overlay) and ATG7 KD (blue overlay) cells. A single representative histogram is shown, with mean fluorescence intensity ± SEM presented in graph to the right **(ii)** (n = 4) **p < 0.005.

We have previously demonstrated the importance of the autophagy regulator ATG7, in ATRA-induced differentiation (24). Here we have investigated whether ATG7 is also important for autophagy and differentiation induced by the combination of ATRA and VPA. ATG7 was depleted by lentivirus-mediated shRNA knockdown in NB4 cells, as previously described and validated in (24). Following treatment with either VPA (1 mM) or ATRA (1 µM) alone or in combination for 72 hours, autophagy levels were compared in scrambled (Scr) and the ATG7 KD clone. Basal autophagy was significantly reduced in the ATG7 KD clone [**Figure 3A** **(i)** black overlay] compared to the Scr control cells (grey filled overlay) (p = 0.0031). Triplicate data is graphed in **Figure 3A** **(ii)**. In addition, the loss of ATG7 completely reduced autophagy induced by the VPA and ATRA combination (blue overlay), compared to Scr control cells (red overlay) (p < 0.0001). The loss of ATG7 also significantly reduced ATRA-induced and VPA-induced autophagy [**Supplementary Figure 4A (i, ii)**, pink and blue overlay respectively]. Western blot analysis of LC3II confirmed these data, with significant reduction in LC3II levels in ATRA and combination treated ATG7 KD clones (**Figure 3B**, lanes 4 and 8).

The loss of ATG7 however, induced a significant increase in basal expression of surface CD11β [**Figure 3C** **(i, ii)** black overlay] compared to Scr control cells (grey filled overlay) (p = 0.0074). Despite this increase in basal expression, VPA and ATRA-induced CD11β expression was significantly reduced in the ATG7 clone (blue overlay) (p = 0.0014) compared to the scramble control treated clone (red overlay). The loss of ATG7 also attenuated ATRA-induced CD11β expression [**Supplementary Figure 4B (i, ii)** pink overlay] with no change in VPA-induced expression [**Supplementary Figure 4B (i, ii)**, blue overlay]. We cannot rule out the possibility that autophagy may be involved in the stability or degradation of CD11β. Expression of CD11β might then be elevated by depletion of ATG7. Nevertheless, loss of ATG7 still reduces the significant enhancement of CD11β expression induced by the ATRA VPA combination treatment (blue and red histogram overlays) suggesting that autophagy plays a role in the promotion of differentiation by this treatment.

### shRNA-Mediated Depletion of TFEB Attenuates VPA and ATRA-Induced Autophagy and Differentiation in APL Cells

**Figure 4:**
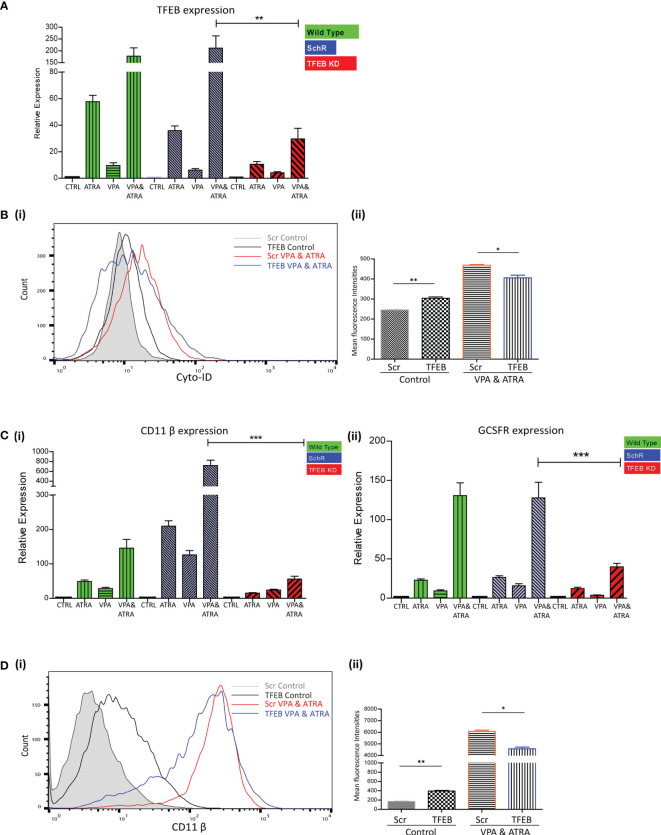
TFEB knockdown attenuated valproic acid (VPA) & all-*trans*-retinoic acid (ATRA)-induced autophagy and differentiation. TFEB was knocked down in NB4 cells using lentiviral transduction of target-specific short hairpin (sh)RNA (TFEB). NB4 cells were also transduced with an off-target scrambled shRNA (Scr). Both cell lines were treated with either ATRA (1 μM) or VPA (1mM) alone or a combination of both for 72 hours. **(A)** Expression levels of TFEB were assessed by RT-qPCR, in wild type (green bars), Scr (blue bars) and TFEB KD (red bars) cell lines. Raw Ct values were normalised to a housekeeping gene and data are shown as n-fold induction compared to untreated controls for each cell line (n = 3) **p < 0.005. **(B) (i)** Cyto-ID was used to assess autophagosome formation in untreated Scr (grey histogram), untreated TFEB knockdown (black overlay), VPA&ATRA treated Scr (red overlay) and TFEB knockdown (blue overlay) cell lines. **(ii)** Data from three independent experiments is presented in the graph to the right, as mean fluorescence intensities ± SEM **p < 0.005, *p < 0.05. **(C)** Expression levels of known differentiation genes CD11β **(i)** and GCSFR **(ii)** were assessed by RT-qPCR, in wild type (green bars) Scr (blue bars) and TFEB KD (red bars) cells. Raw Ct values were normalised to a housekeeping gene and data are shown as n-fold induction compared to untreated controls for each cell line (n = 3) ***p < 0.0005. **(D) (i)** The induction of differentiation was assessed by measuring expression of surface CD11β by flow cytometry in untreated Scr (grey histogram), untreated TFEB KD (black overlay), and VPA&ATRA treated Scr (red overlay) and TFEB KD (blue overlay) cells. A single representative histogram is shown, with mean fluorescence intensity ± SEM presented in the graph to the right **(ii)** (n = 4) **p < 0.005, *p < 0.05.

We have previously demonstrated the significant role that TFEB plays in ATRA-induced autophagy and differentiation (25). We therefore investigated whether TFEB is also involved in the autophagy and granulocytic differentiation induced by the combination treatment in NB4 cells. As previously described (25), TFEB was depleted by lentivirus-mediated shRNA knockdown in NB4 cells. Following treatment with either VPA or ATRA alone or in combination for 72 hours, expression levels of TFEB were assessed by RT-qPCR, in wild type (green bars), scrambled control (Scr) (blue bars) and a TFEB KD clone (red bars). A significant reduction of TFEB expression was evident in all treatments of the TFEB KD clone (red histograms), relative to the Scr and Wild Type (**Figure 4A**) (p = 0.0013). Expression levels of additional transcripts, identified as important for autophagy and induced by ATRA, in our previous RNAseq analysis were also examined (25). *ATG16L*, *CTSD* and *GABARAP* mRNA levels were all reduced in the TFEB KD clone following treatment [**Supplementary Figure 5A (i-iii)**].

Unlike the ATG7 KD, the loss of TFEB induced an increase in basal autophagosome levels (black overlay) compared to Scr controls (filled grey overlay) (p = 0.0088). However, loss of TFEB still significantly reduced the autophagy induced by the combination of VPA & ATRA (blue overlay) compared to the Scr control treated clone (red overlay) [**Figure 4B (i, ii)**] (p = 0.049). The loss of TFEB also reduced ATRA-induced and VPA-induced autophagy [**Supplementary Figure 5B (i, ii)**, pink and blue overlay respectively].

Expression levels of *CD11β* and *GCSFR* as determined by RT-qPCR, again showed a marked reduction in the TFEB knockdown clone [**Figure 4C** **(i, ii)** red bars], following treatment with ATRA, VPA or the combination (p < 0.0001, 0.0001).

Similar to ATG7 knockdown clones, the loss of TFEB induced a significant increase in basal expression of surface CD11β (black overlay) compared to scramble controls (grey histogram) [**Figure 4D** **(i, ii)**] (p = 0.0027). Yet, again there was still a significant reduction in the VPA & ATRA-induced CD11β expression (blue overlay) in the TFEB knockdown clone, as compared to the Scr control treated clone (red overlay) (p = 0.0231). The loss of TFEB also significantly reduced the ATRA-induced CD11β expression [**Supplementary Figure 5C (i, ii)**, pink overlay] with an increase in VPA-induced expression [**Supplementary Figure 5C (i, ii)**, blue overlay].

Together, these data demonstrate that loss of either autophagy regulators, ATG7 or TFEB, impedes granulocytic differentiation induced by ATRA and VPA co-treatment, demonstrating the importance of autophagy in the activity of this combination.

### Brefeldin A (BFA) Augmented the Effects of ATG7 and TFEB Depletion on VPA and ATRA-Induced Autophagy and Differentiation in APL Cells

TFEB is only one of several transcription factors known to influence autophagy and alternative mechanisms of autophagy have been described which do not require all of the canonical components of autophagy initiation complexes (16). We previously showed that ATRA could induce autophagy that was independent of TFEB (25). This alternative ATRA-induced autophagy was sensitive to the Golgi inhibitor BFA, which disrupts an autophagy pathway that originates at the Golgi (17). We therefore examined if BFA-sensitive autophagy might contribute to the autophagy induced by the combination of ATRA and VPA in the ATG7 and TFEB KD cells. Scramble, ATG7 and TFEB KD clones were treated with ATRA (1 µM) and VPA (1 mM) for 72 hours, and 24 hours prior to analysis, BFA (10 µM) was added to inhibit Golgi-initiated autophagy.

**Figure 5:**
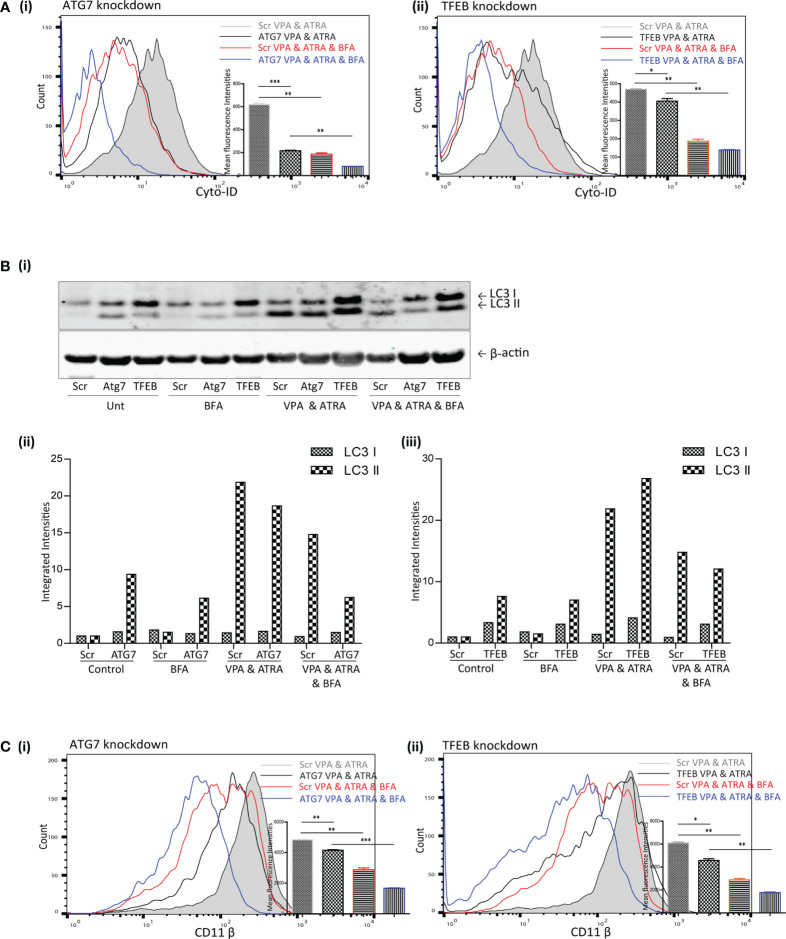
Brefeldin A (BFA) augmented the effects of ATG7 and TFEB depletion on VPA & ATRA-induced autophagy and differentiation. NB4 cells expressing non-targeting shRNA (Scr) or shRNA targeting ATG7 or TFEB were treated with a combination of VPA (1mM) & ATRA (1 μM) for 72 hours. To assess the effects of BFA on VPA&ATRA-induced autophagy and differentiation, cells were treated with BFA (10 μM) for 24 hours prior to analysis. **(A)** Cyto-ID was used to assess autophagosome formation in ATG7 knockdown **(i)** or TFEB knockdown **(ii)** cell lines; VPA&ATRA treated Scr (grey histograms), VPA&ATRA treated knockdown clones (black overlays), VPA&ATRA&BFA treated Scr (red overlays) and knockdown clones treated with VPA&ATRA&BFA (blue overlays). Data from three independent experiments is presented in the inset graph, as mean fluorescence intensities ± SEM ***p < 0.0005, **p < 0.005, *p < 0.05. **(B)** Western blot analysis of LC3II levels in Scr, ATG7 and TFEB knockdown cells, following treatment with VPA and ATRA in the absence or presence of BFA **(i)**. All bands were quantified using the Odyssey Infrared Imaging System (Li-COR), normalised to ß-actin and presented here as separate integrated intensities for each knockdown cell line – Atg7 **(ii)** and TFEB **(iii)**, with all bands normalised to Scr untreated control cells (lane 1). **(C)** The induction of differentiation was assessed by measuring expression of surface CD11β in in ATG7 knockdown **(i)** or TFEB knockdown **(ii)** cell lines; VPA&ATRA treated Scr (grey histograms), VPA&ATRA treated knockdown clones (black overlays), VPA&ATRA&BFA treated Scr (red overlays) and knockdown clones treated with VPA&ATRA&BFA (blue overlays). Data from three independent experiments is presented in the inset graph, as mean fluorescence intensities ± SEM ***p < 0.0005, **p < 0.005, *p < 0.05.

The addition of BFA to the combination treated scramble cells (Scr VPA&ATRA, red overlay) significantly reduced autophagosome accumulation relative to combination treated Scr cells (grey histogram) (p = 0.0026 and p = 0.001, respectively) [**Figure 5A** **(i, ii)**)] Knockdown of ATG7 or TFEB also significantly reduced the level of autophagosomes induced by the combination treatment of VPA and ATRA (black overlays) relative to their scramble control cells (grey histogram) (p < 0.0001 and p = 0.049, respectively). Importantly, autophagosome levels were then further reduced by the addition of BFA in both Atg7 (p = 0.0012) and TFEB (p = 0.0017) knockdown clones (**Figure 5A** blue overlays versus black). 

LC3II levels were assessed for the same treatments by Western blot analysis [**Figure 5B** **(i)**]. These are graphed separately [**Figure 5B** **(ii, iii)**] for each knockdown cell line for clarity. These data indicate that the addition of BFA significantly reduced LC3II levels in VPA & ATRA treated clones [**Figure 5B** **(ii, iii)**]. It has been reported that LC3II is not necessarily required for alternative autophagy (16, 18), but here we notice an impact on LC3II in response to the presence of BFA. This may be a result of an overlap between pathways or effects of BFA on overall protein synthesis.

Disruption of this alternative, Golgi-derived autophagy with BFA resulted in a corresponding reduction of VPA&ATRA-induced differentiation in both clones [**Figure 5C** **(i, ii)**, blue overlays versus black] (p = 0.0005 and p = 0.0027 respectively).

These data were combined as triplicate mean fluorescence intensities, normalised to combination treated scramble clones. Taken together these data highlight the effect of ATG7 and TFEB silencing on VPA&ATRA-induced autophagy and differentiation, an effect that is further enhanced by BFA treatment. These experiments do not allow us to differentiate between a reduction in surface CD11β due to inhibition of an alternative autophagy pathway or due to impaired Golgi trafficking. More selective inhibitors of alternative autophagy pathways should help to address this question in future studies.

## Discussion

This study has shown that VPA can promote autophagy and differentiation in ATRA treated APL cells and in ATRA resistant APL and non-APL myeloid leukemia cells. Autophagy is important for differentiation as shRNA knockdown of the key autophagy regulators, ATG7 or TFEB, impedes both autophagy and differentiation. The mechanism by which autophagy is induced by VPA is currently unknown and may be related to its activity as a HDAC inhibitor.

As HDAC enzymes contribute to the repressive effects of PML-RARα, a combination of a ATRA and a HDAC inhibitor is a logical approach to improve efficacy. VPA has shown activity in inducing apoptosis, arrest or differentiation in a variety of cell line models (reviewed in (27) and primary AML blasts (35). Several phase I/II clinical trials have been conducted in the last decade with myelodysplastic syndrome (MDS) or AML using VPA as a monotherapy or in combination with ATRA. Activity was modest and disappointing overall, as complete or partial remissions were rarely observed [reviewed in (27)]. Trials with other HDAC agents as single agents have also shown modest hematologic improvements in a subset of patients (15). Further combination strategies to improve efficacy have been evaluated, such as combining a demethylating agent to help release transcriptional repression. A recent Phase I/II trial combined 5-azacytidine with VPA and ATRA, with impressive results. The overall response rate was 42% and in previously untreated older patients, the response rate was 52% which is favourable for an out-patient based therapy (36); (https://clinicaltrials.gov/ct2/show/NCT00326170). These data compare advantageously to trials with 5-aza-2’-deoxycytidine (decitabine) and 5-azacytidine as single agents, but a randomised trial would be required to establish this. Four biomarkers were evaluated in this trial: VPA levels, histone acetylation, global DNA hypomethylation and induction of p21 and p15 expression. Higher VPA levels were found in patients who responded, versus those who did not, which implies that a more potent HDAC inhibitor may improve the activity of the protocol. However, while histone acetylation was observed, this was not associated with response, nor were VPA levels associated with histone acetylation. In addition, there was no correlation between clinical response and induction of hypomethylation, or expression of *p21* and *p15* mRNA. Other molecular effects may therefore contribute to the efficacy of this drug combination in these patients.

Several studies have now demonstrated that acetylation regulates many non-histone targets (37). Class II HDAC inhibitors have been shown to target p53 and HIF1α and have cytoplasmic targets, including a-tubulin, HSP70 and HSP90 [reviewed in (15)]. Analysis of VPA targets in CML cells identified several acetylated cytoplasmic proteins including HSP90 and hnRNPL (38). Recent advances in mass spectrometry proteomic approaches have identified thousands of novel acetylation sites, with most on non-nuclear proteins. Acetylation was particularly widespread in the mitochondrial proteome and metabolic enzymes (39). It is therefore possible that the key targets of successful HDAC inhibitors have yet to be discovered and current markers of activity are therefore unable to represent efficacy of these agents.

Our data suggest that autophagy is important for the differentiation induced by the combination of VPA and ATRA. Autophagy has previously been shown to be induced by VPA, but the mechanism is poorly understood. It is possible that autophagy genes are epigenetically silenced in these cells and this is relieved by VPA. Epigenetic regulation of autophagy has been well described (40, 41). However, it is also possible that autophagy is promoted by non-epigenetic mechanisms. Acetylation of cytoplasmic autophagy regulators ULK1, TFEB and ATG proteins has been reported (42–44). It is also interesting that tubulin acetylation is essential for fusion of autophagosomes to lysosomes (45). A HDACi may therefore improve this trafficking of autophagosomes to lysosomes and therefore reduce the overall cellular content of autophagosomes, due to more efficient turnover. This is also consistent with an unexplained feature in **Figure 2B** **(i)**, where VPA reduced the basal autophagosome content (measured with Cyto-ID), an effect that was abolished by inhibiting the lysosome with chloroquine, **Figure 2B** **(ii)**. Further studies are now required to properly evaluate the key targets of VPA and other HDAC inhibitors, so that better markers for clinical trials can be established. Our data would suggest that where induction of differentiation is the objective of treatment, a marker of autophagy induction could be useful.

Our data with BFA indicates that non-canonical/alternative autophagy may also play a significant role in differentiation in AML cells. Further knockdown studies with genes specific for this pathway would be needed to confirm this. Interestingly, another study has suggested that haematopoietic stem cells rely on ATG7-dependant canonical autophagy, whereas more differentiated myeloid cells can use either pathway (46). Beclin1 independent autophagy has also been recently reported during ATRA induced differentiation of APL cells (23). Undoubtedly further insights are into the diverse mechanisms of autophagy regulation and the importance of specific pathways are required so that autophagy can be more selectively modulated for clinical benefit.

The Bcl-2 inhibitor Venetoclax has recently emerged as a new treatment option for older adults with AML and may become the benchmark for testing new developments. As a single agent it has modest anti-leukemic activity (47), however combinations with hypomethylating agents (HMAs) azacitidine/decitabine or with low-dose cytarabine (LDAC) have shown activity and were FDA approved in 2018 for AML [reviewed in (48)]. Further studies are however required on the molecular determinants of response. Venetoclax is only modestly efficacious in relapsed/refractory or secondary AML and AML patients with adverse cytogenetics and high risk mutations continue to have poorer outcomes [reviewed in (49)]. The full extent of its mechanisms of action, including possible actions on autophagy, remain to be defined. The expression levels of Bcl-2 alone do not seem to be predictive of response, rather the expression of interacting Bcl-2 family members, such as Bax and Bad and upregulation of other anti-apoptotic family members such as Mcl-1 and Bcl-xL are thought to be more relevant (47, 49). It is notable that one of the key interacting partners of Bcl-2 is the autophagy regulator Beclin-1. Beclin-1 has a BH3 domain that can interact with Bcl-2 or Bcl-xL (50). It is interesting in this regard that many BH3 mimetics have been shown to induce autophagy (51, 52), including venetoclax (53, 54). It would be interesting to assess whether venetoclax can influence autophagy and ATRA induced differentiation in AML cells.

The overall concept of differentiation therapy is particularly apposite given recent experience with the global COVID-19 pandemic, when hospital resources have never been scarcer and the need to avoid profound immunosuppression in patients with blood cancers is a high priority. Re-examination of inexpensive, low toxicity compounds such as VPA is more relevant now than ever before. It is hoped that new strategies will emerge for the potential treatment of AML patients, particularly aimed at reducing the complexity of clinical care and improving quality of life.

## Conclusions

This study has shown that a combination of valproic acid and ATRA can induce differentiation in myeloid leukemia cells. shRNA knockdown of ATG7 or TFEB autophagy regulators impaired both autophagy and differentiation, indicating the importance of autophagy in this combination treatment. In addition, impeding non-canonical/alternative autophagy with brefeldin A (BFA), was found to further inhibit VPA and ATRA induced autophagy and differentiation in the knockdown cells, suggesting the involvement of more than one autophagy pathway. These data support accumulating evidence that autophagy is a key component of an effective differentiation-inducing regimen in myeloid leukemia cells.

Other clinical studies would suggest that additional compounds are likely to be needed to comprise a clinically efficacious regimen. Our data would suggest that new combination strategies should consider the impact of modulation of autophagy on the compounds being tested. Further interrogation of the mechanistic interplay between autophagy pathways and differentiation in leukemia is warranted to improve therapeutic regimes in the future

## Data Availability Statement

The original contributions presented in the study are included in the article/**Supplementary Material**. Further inquiries can be directed to the corresponding author.

## Author Contributions

Conceptualization; SLM, LJG, NPM, TOD and MRC. Methodology; TOD, DNB, KBL, NO. Data curation and analysis, TOD, DNB, KBL. Construction and validation of ATG7 and TFEB knockdown cell lines, NO and TOD. Preparation of manuscript; SLM and TOD. Review and editing; SLM, TOD, MRC, LJG, KBL and NM. Supervision, SLM, LJG, and NPM. All authors have read and agreed to the published version of the manuscript.

## Funding

DB was funded by the Haematology Education and Research Trust (HERO) and Breakthrough Cancer Research (BCR). TO’D was funded by BCR. This research was also supported by the National Institutes of Health (CA043796) (LG.) and by Weill Cornell funds. The financial support of the University of Nottingham, University College Cork and Higher Education Authority of Ireland is gratefully acknowledged.

## Conflict of Interest

The authors declare that the research was conducted in the absence of any commercial or financial relationships that could be construed as a potential conflict of interest.

## Publisher’s Note

All claims expressed in this article are solely those of the authors and do not necessarily represent those of their affiliated organizations, or those of the publisher, the editors and the reviewers. Any product that may be evaluated in this article, or claim that may be made by its manufacturer, is not guaranteed or endorsed by the publisher.

## Supplementary Material

Supplementary Figure 1 VPA promotes ATRA-mediated myeloid differentiation in ATRA sensitive and ATRA resistant, acute promyelocytic leukemia (APL) cell lines (NB4 and NB4R). NB4 and NB4R cell lines were treated with either ATRA (1 μM) or VPA (1mM) alone or a combination of both for 72 hours. The induction of differentiation was assessed by measuring expression levels of additional differentiation genes CEBP and ID2 by RT-qPCR. Raw Ct values were normalised to a housekeeping gene and data are shown as n-fold induction compared to untreated controls (n = 3). Expression levels of ID2 were significantly enhanced by the addition of VPA to ATRA in the ATRA-resistant NB4R cells. CEBPε was not significantly induced in NB4R cells relative to ATRA alone. Expression levels were not increased in ATRA-sensitive NB4 cells – which are already sensitive to ATRA. ***p < 0.0005, **p < 0.005, *p < 0.05.

Supplementary Figure 2 Valproic acid (VPA) promotes all-*trans*-retinoic acid (ATRA)-mediated differentiation and autophagy in ATRA resistant, non-APL THP-1 cells. The THP-1 cell line was treated with either ATRA (1 μM) or VPA (1mM) alone or a combination of both for 72 hours. Induction of differentiation was assessed by measuring **(A)** expression levels of known differentiation genes **(i)** CD11β and **(ii)** GCSFR by RT-qPCR. Raw Ct values were normalised to a housekeeping gene and data are shown as n-fold induction compared to untreated controls (n = 3). **(B)** Expression of surface CD11β by flow cytometry was assessed. Control untreated (grey histogram), ATRA (red overlay), VPA (green overlay) and VPA&ATRA treated cells (blue overlay). A single representative histogram is shown, with mean fluorescence intensity ± SEM presented in the graph to the right (n = 4). **(C)** Morphological features of granulocytic differentiation were assessed by light microscopy (400x) in THP-1 cells. Red arrows indicate cells with features of granulocytic differentiation. Autophagy induction was assessed by measuring **(D)** expression levels of genes, important for autophagy, **(i)** TFEB and **(ii)** ATG16L by RT-qPCR. Raw Ct values were normalised to a housekeeping gene and data are shown as n-fold induction compared to untreated controls (n = 3). **(E) (i)** Cyto ID was used to assess autophagosome formation in response to ATRA (red overlay), VPA (green overlay) and VPA&ATRA (blue overlay) relative to untreated controls (grey histogram). **(ii)** Flux was analysed with the addition of CQ to treated cells, relative to CQ alone control cells (grey histogram). Data from three independent experiments is presented in the inset graph as mean fluorescence intensities ± SEM. **(iii)** Western blot analysis of LC3II levels in the absence (lanes 1-4) and presence of chloroquine (CQ) (lanes 5-8). An increase in LC3II in cells treated with VPA&ATRA & CQ (lane 8), beyond that induced with chloroquine alone (lane 5) is indicative of flux. All bands were quantified using the Odyssey Infrared Imaging System (Li-COR), normalised to ß-actin and presented as integrated intensities, with all bands normalised to untreated control cells (lane 1) ***p < 0.0005, **p < 0.005, *p < 0.05. For all western blots, integrated intensities are representative of three independent experiments (n = 3).

Supplementary Figure 3 Valproic acid (VPA) promotes all-*trans*-retinoic acid (ATRA)-mediated autophagy in ATRA sensitive and ATRA resistant, acute promyelocytic leukemia (APL) cell lines (NB4 and NB4R). NB4 and NB4R cell lines were treated with either ATRA (1μM) or VPA (1mM) alone or a combination of both for 72 hours. Induction of autophagy was assessed by measuring expression levels of additional autophagy genes GABARAP and CTSD by RT-qPCR. Raw Ct values were normalised to a housekeeping gene and data are shown as n-fold induction compared to untreated controls (n = 3). Expression levels of both additional markers were enhanced by the addition of VPA to ATRA in the ATRA-resistant NB4R cells. Levels were not significantly different from ATRA alone in the ATRA-sensitive NB4 cells ***p < 0.0005, **p < 0.005.

Supplementary Figure 4 ATG7 knockdown attenuated valproic acid (VPA) and all-*trans*-retinoic acid (ATRA)-induced autophagy and acute promyelocytic leukemia (APL) cell differentiation. ATG7 was knocked down in NB4 cells using lentiviral transduction of target-specific short hairpin (sh)RNA (ATG7). NB4 cells were also transduced with an off-target scrambled shRNA (Scr). Both cell lines were treated with either ATRA (1μM) or VPA (1mM) alone for 72 hours. **(A) (i)** Cyto-ID was used to assess autophagosome formation in control untreated Scr (grey histogram), untreated ATG7 knockdown (black overlay), ATRA treated Scr (red overlay) and ATG7 knockdown (pink overlay) and VPA treated Scr (green overlay) and ATG7 knockdown (blue overlay) cell lines. **(ii)** Data from three independent experiments is presented in the graph to the right, as mean fluorescence intensities ± SEM. **(B) (i)** Expression of surface CD11β was assessed in control untreated Scr (grey histogram), untreated ATG7 knockdown (black overlay), ATRA treated Scr (red overlay) and ATG7 knockdown (pink overlay) and VPA treated Scr (green overlay) and ATG7 knockdown (blue overlay) cell lines. A single representative histogram is shown, with mean fluorescence intensity ± SEM presented in graph to the right (n = 4).

Supplementary Figure 5 TFEB knockdown attenuated VPA and ATRA-induced autophagy and differentiation. TFEB was knocked down in NB4 cells using lentiviral transduction of target-specific short hairpin (sh)RNA (TFEB). NB4 cells were also transduced with an off-target scrambled shRNA (Scr). Both cell lines were treated with either ATRA (1μM) or VPA (1mM) alone for 72 hours. **(A)** Expression levels of additional genes, identified as important for autophagy were examined. Expression levels of **(i)** ATG16L, **(ii)** CTSD and **(iii)** GABARAP were reduced in the TFEB clone, following treatment (red bars). **(B) (i)** Cyto-ID was used to assess autophagosome formation in control untreated Scr (grey histogram), untreated TFEB knockdown (black overlay), ATRA treated Scr (red overlay) and TFEB knockdown (pink overlay) and VPA treated Scr (green overlay) and TFEB knockdown (blue overlay) cell lines. **(ii)** Data from three independent experiments is presented in the graph to the right, as mean fluorescence intensities ± SEM (* **). **(C) (i)** Expression of surface CD11β was assessed. Control untreated Scr (grey histogram), untreated TFEB knockdown (black overlay), ATRA treated Scr (red overlay) and TFEB knockdown (pink overlay) and VPA treated Scr (green overlay) and TFEB knockdown (blue overlay) cell lines. A single representative histogram is shown, with mean fluorescence intensity ± SEM presented in graph to the right (n = 4) ***p < 0.0005, **p < 0.005, *p < 0.05.

## Abbreviations

AML, acute myeloid leukaemia; APL, acute promyelocytic leukaemia; ATG, autophagy related; ATO, arsenic trioxide; ATRA, All-*trans*-retinoic acid; BECN1, BCL-2-interacting coiled-coil protein or Beclin 1; BFA, brefeldin A; CEPBe, CCAAT enhancer-binding protein; CQ, chloroquine; CTSD, cathepsin D; ER, endoplasmic reticulum; GABARAP, gamma-aminobutyric-acid-type-A-receptor-associated protein; GCSFR, granulocyte colony-stimulating factor receptor; HATS, histone acetyltransferases; HDAC, histone deacetylase; HDACi, histone deacetylase inhibitor; hnRNPL, heterogeneous nuclear ribonucleoprotein L; HPRT, hypoxanthine phosphorybosyl transferase; HSP, heat shock protein; HSPC, heamatopoietic stem and progenitor cells; ID2, inhibitor of DNA binding family member; LC3, microtubule-associated protein 1α/β-light chain 3; LDAC, low-dose cytarabine; MDS, Myelodysplastic syndrome; PE, phosphatidylethanolamine; RARE’s, retinoic acid response elements. Scr, off-target scrambled shRNA; TFEB, transcription factor EB; VPA, valproic acid.
