# Supplementary figures and images for: All-Trans-Retinoic Acid Combined With Valproic Acid Can Promote Differentiation in Myeloid Leukemia Cells by an Autophagy Dependent Mechanism
Source: Front Oncol. 2022 Feb 24;12:848517. doi: 10.3389/fonc.2022.848517 (PMC8907478; doi:10.3389/fonc.2022.848517)

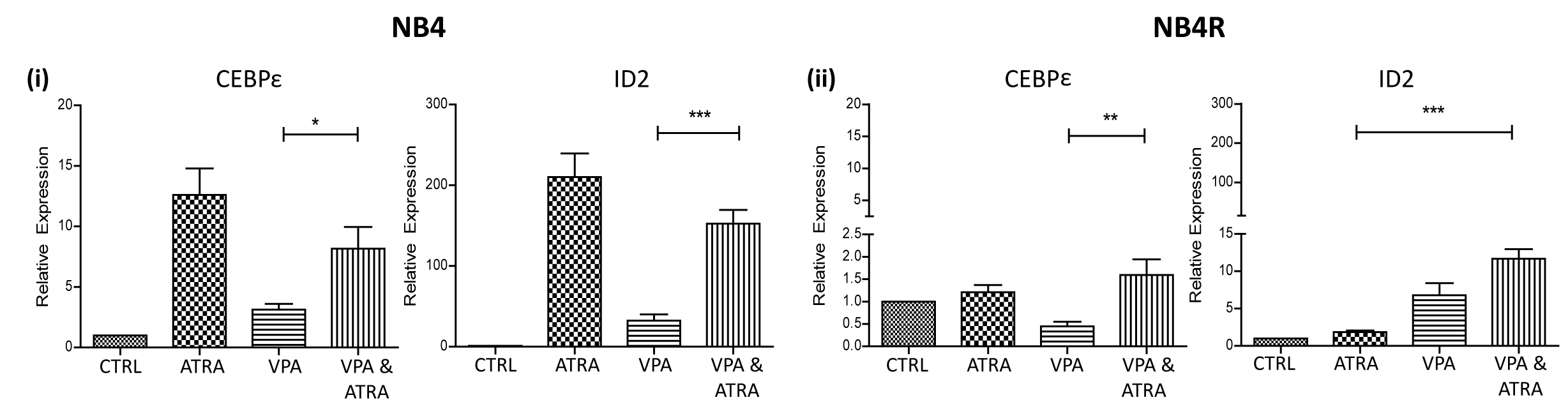

Supplement: Supplementary Figure 1 — VPA promotes ATRA-mediated myeloid differentiation in ATRA sensitive and ATRA resistant, acute promyelocytic leukemia (APL) cell lines (NB4 and NB4R). NB4 and NB4R cell lines were treated with either ATRA (1 μM) or VPA (1mM) alone or a combination of both for 72 hours. The induction of differentiation was assessed by measuring expression levels of additional differentiation genes CEBP and ID2 by RT-qPCR. Raw Ct values were normalised to a housekeeping gene and data are shown as n-fold induction compared to untreated controls (n = 3). Expression levels of ID2 were significantly enhanced by the addition of VPA to ATRA in the ATRA-resistant NB4R cells. CEBPε was not significantly induced in NB4R cells relative to ATRA alone. Expression levels were not increased in ATRA-sensitive NB4 cells – which are already sensitive to ATRA. ***p < 0.0005, **p < 0.005, *p < 0.05. [file Image_1.tif]

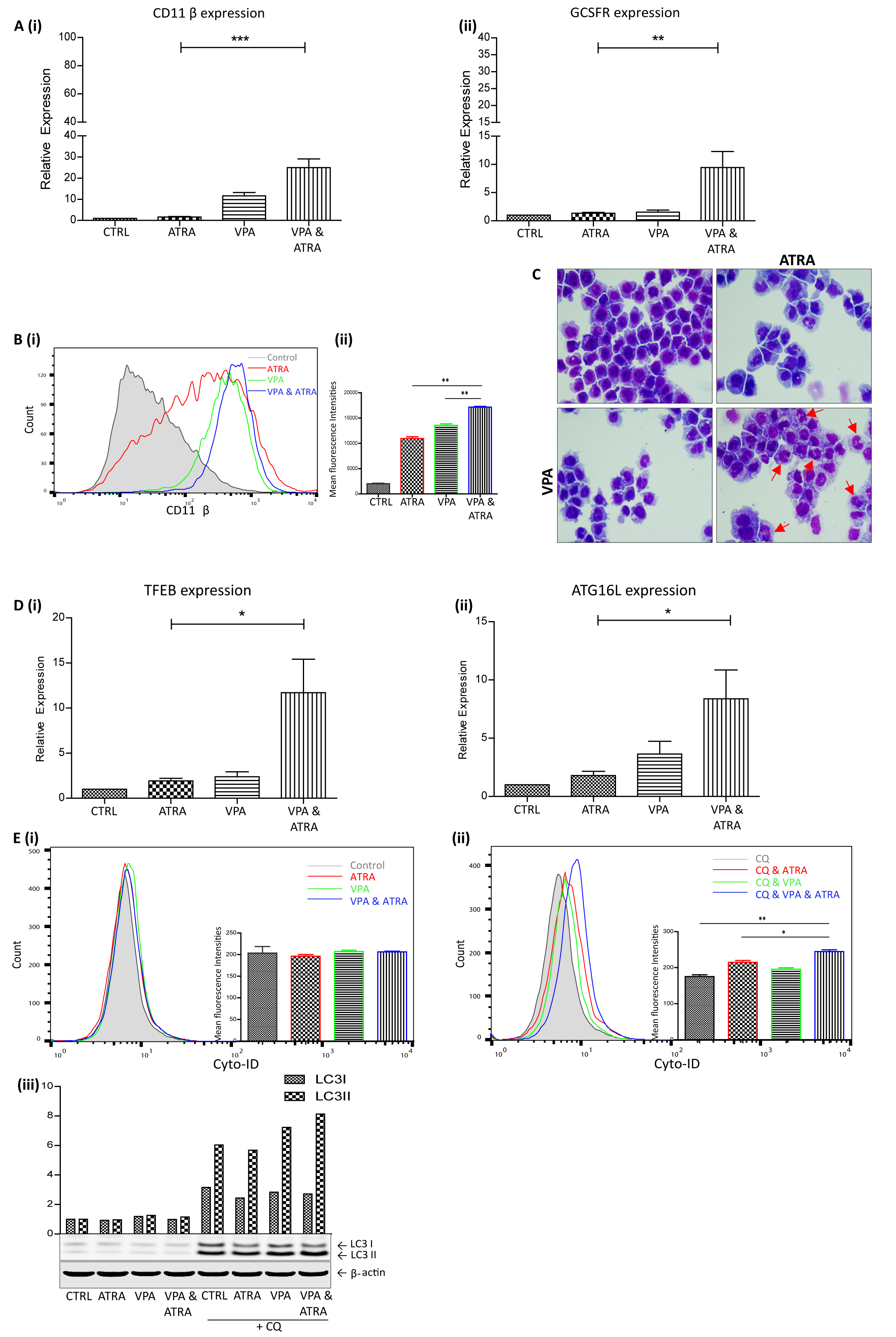

Supplement: Supplementary Figure 2 — Valproic acid (VPA) promotes all-trans-retinoic acid (ATRA)-mediated differentiation and autophagy in ATRA resistant, non-APL THP-1 cells. The THP-1 cell line was treated with either ATRA (1 μM) or VPA (1mM) alone or a combination of both for 72 hours. Induction of differentiation was assessed by measuring (A) expression levels of known differentiation genes (i) CD11β and (ii) GCSFR by RT-qPCR. Raw Ct values were normalised to a housekeeping gene and data are shown as n-fold induction compared to untreated controls (n = 3). (B) Expression of surface CD11β by flow cytometry was assessed. Control untreated (grey histogram), ATRA (red overlay), VPA (green overlay) and VPA&ATRA treated cells (blue overlay). A single representative histogram is shown, with mean fluorescence intensity ± SEM presented in the graph to the right (n = 4). (C) Morphological features of granulocytic differentiation were assessed by light microscopy (400x) in THP-1 cells. Red arrows indicate cells with features of granulocytic differentiation. Autophagy induction was assessed by measuring (D) expression levels of genes, important for autophagy, (i) TFEB and (ii) ATG16L by RT-qPCR. Raw Ct values were normalised to a housekeeping gene and data are shown as n-fold induction compared to untreated controls (n = 3). (E) (i) Cyto ID was used to assess autophagosome formation in response to ATRA (red overlay), VPA (green overlay) and VPA&ATRA (blue overlay) relative to untreated controls (grey histogram). (ii) Flux was analysed with the addition of CQ to treated cells, relative to CQ alone control cells (grey histogram). Data from three independent experiments is presented in the inset graph as mean fluorescence intensities ± SEM. (iii) Western blot analysis of LC3II levels in the absence (lanes 1-4) and presence of chloroquine (CQ) (lanes 5-8). An increase in LC3II in cells treated with VPA&ATRA & CQ (lane 8), beyond that induced with chloroquine alone (lane 5) is indicative of flux. All bands were qua [file Image_2.tif]

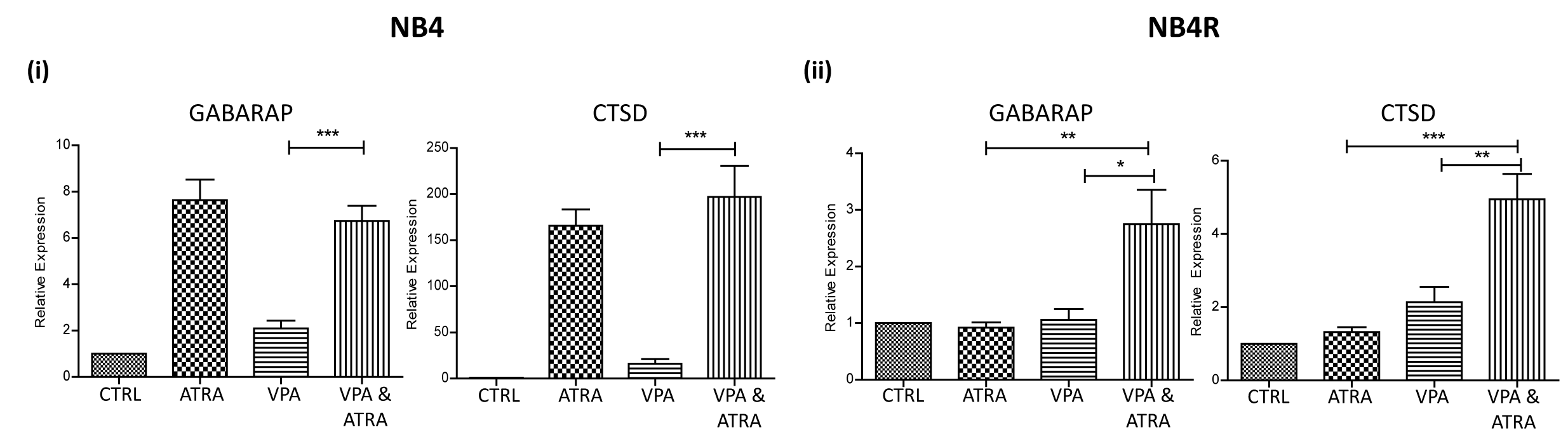

Supplement: Supplementary Figure 3 — Valproic acid (VPA) promotes all-trans-retinoic acid (ATRA)-mediated autophagy in ATRA sensitive and ATRA resistant, acute promyelocytic leukemia (APL) cell lines (NB4 and NB4R). NB4 and NB4R cell lines were treated with either ATRA (1μM) or VPA (1mM) alone or a combination of both for 72 hours. Induction of autophagy was assessed by measuring expression levels of additional autophagy genes GABARAP and CTSD by RT-qPCR. Raw Ct values were normalised to a housekeeping gene and data are shown as n-fold induction compared to untreated controls (n = 3). Expression levels of both additional markers were enhanced by the addition of VPA to ATRA in the ATRA-resistant NB4R cells. Levels were not significantly different from ATRA alone in the ATRA-sensitive NB4 cells ***p < 0.0005, **p < 0.005. [file Image_3.tif]

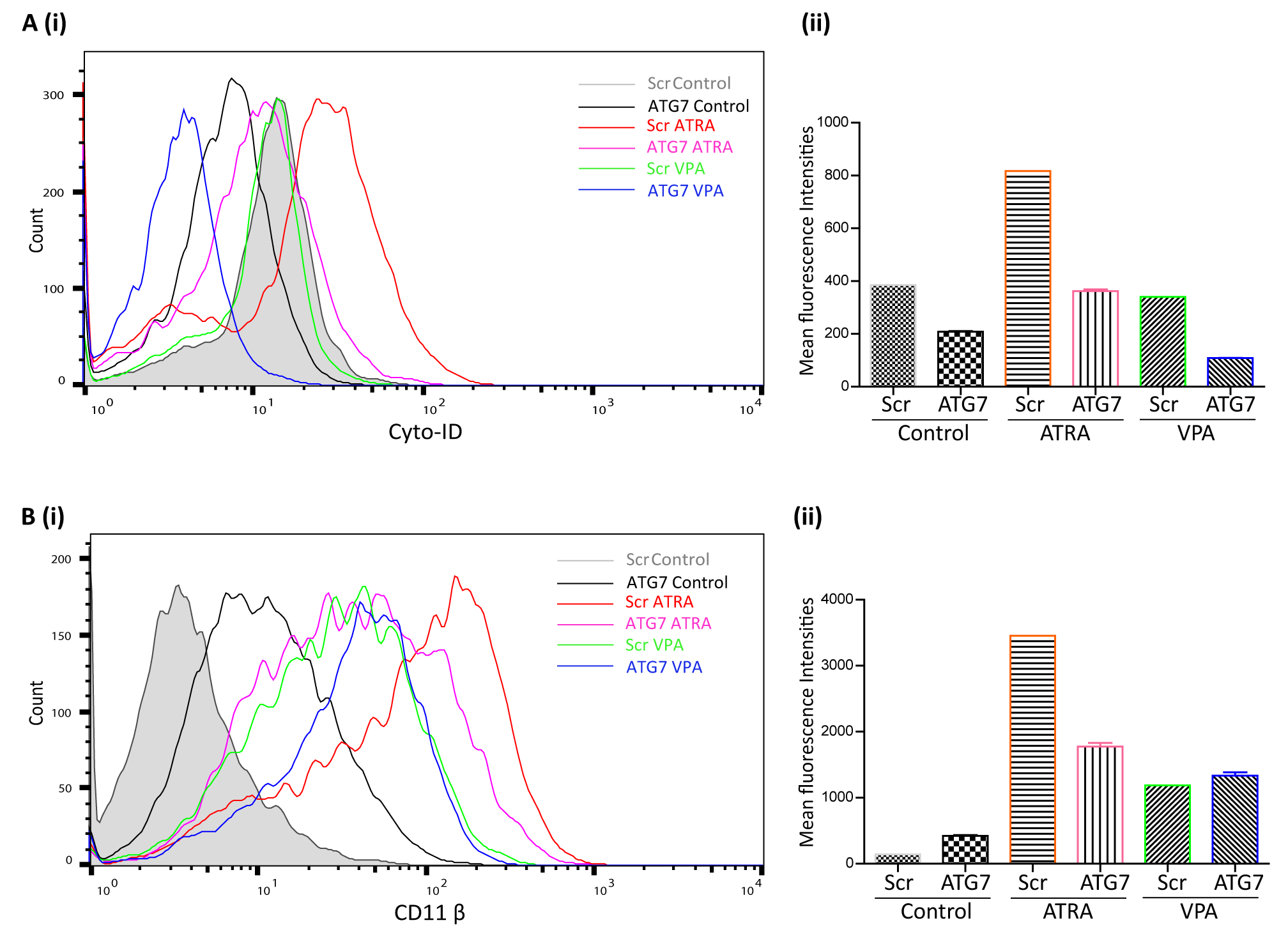

Supplement: Supplementary Figure 4 — ATG7 knockdown attenuated valproic acid (VPA) and all-trans-retinoic acid (ATRA)-induced autophagy and acute promyelocytic leukemia (APL) cell differentiation. ATG7 was knocked down in NB4 cells using lentiviral transduction of target-specific short hairpin (sh)RNA (ATG7). NB4 cells were also transduced with an off-target scrambled shRNA (Scr). Both cell lines were treated with either ATRA (1μM) or VPA (1mM) alone for 72 hours. (A) (i) Cyto-ID was used to assess autophagosome formation in control untreated Scr (grey histogram), untreated ATG7 knockdown (black overlay), ATRA treated Scr (red overlay) and ATG7 knockdown (pink overlay) and VPA treated Scr (green overlay) and ATG7 knockdown (blue overlay) cell lines. (ii) Data from three independent experiments is presented in the graph to the right, as mean fluorescence intensities ± SEM. (B) (i) Expression of surface CD11β was assessed in control untreated Scr (grey histogram), untreated ATG7 knockdown (black overlay), ATRA treated Scr (red overlay) and ATG7 knockdown (pink overlay) and VPA treated Scr (green overlay) and ATG7 knockdown (blue overlay) cell lines. A single representative histogram is shown, with mean fluorescence intensity ± SEM presented in graph to the right (n = 4). [file Image_4.tif]

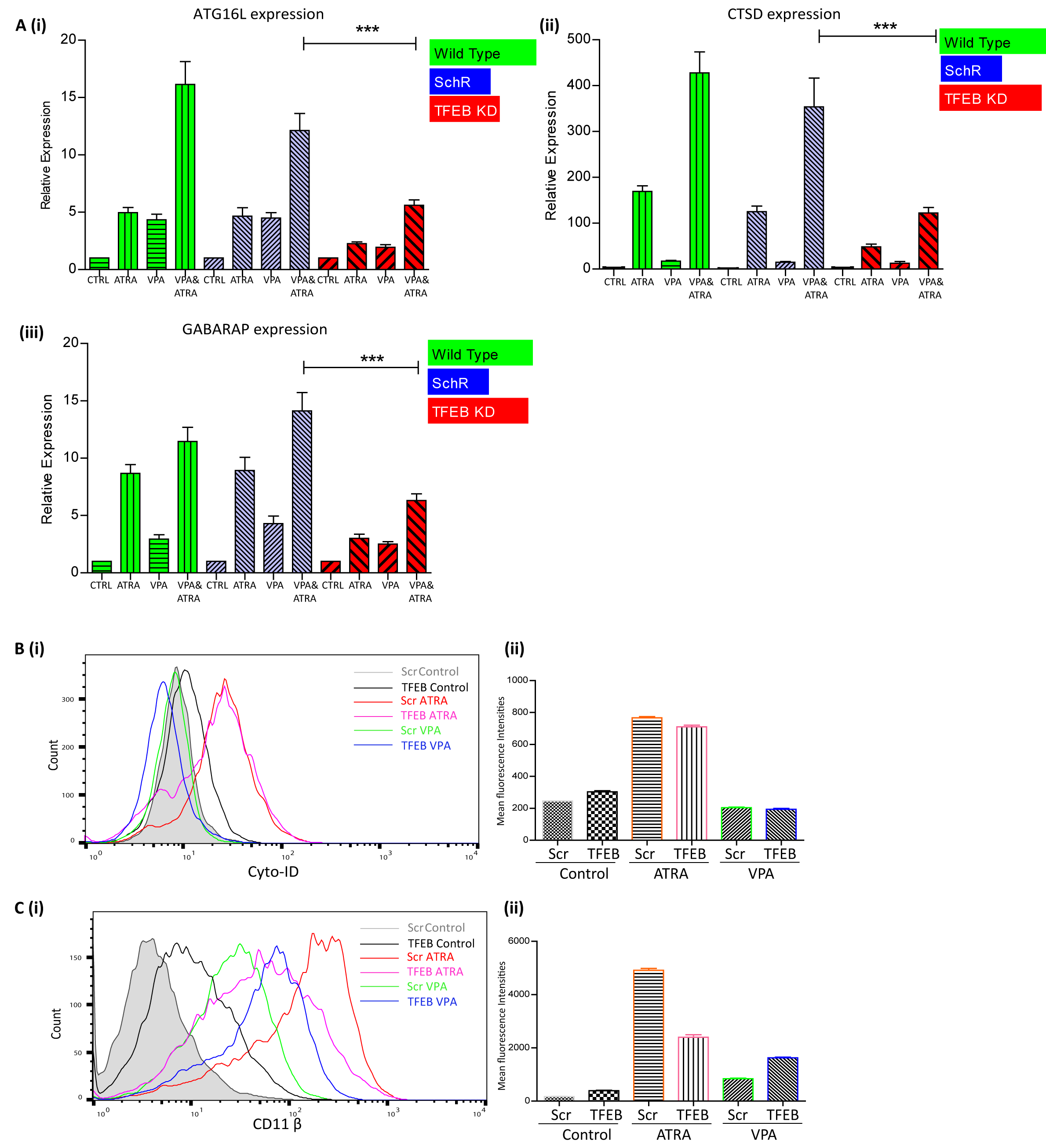

Supplement: Supplementary Figure 5 — TFEB knockdown attenuated VPA and ATRA-induced autophagy and differentiation. TFEB was knocked down in NB4 cells using lentiviral transduction of target-specific short hairpin (sh)RNA (TFEB). NB4 cells were also transduced with an off-target scrambled shRNA (Scr). Both cell lines were treated with either ATRA (1μM) or VPA (1mM) alone for 72 hours. (A) Expression levels of additional genes, identified as important for autophagy were examined. Expression levels of (i) ATG16L, (ii) CTSD and (iii) GABARAP were reduced in the TFEB clone, following treatment (red bars). (B) (i) Cyto-ID was used to assess autophagosome formation in control untreated Scr (grey histogram), untreated TFEB knockdown (black overlay), ATRA treated Scr (red overlay) and TFEB knockdown (pink overlay) and VPA treated Scr (green overlay) and TFEB knockdown (blue overlay) cell lines. (ii) Data from three independent experiments is presented in the graph to the right, as mean fluorescence intensities ± SEM (* **). (C) (i) Expression of surface CD11β was assessed. Control untreated Scr (grey histogram), untreated TFEB knockdown (black overlay), ATRA treated Scr (red overlay) and TFEB knockdown (pink overlay) and VPA treated Scr (green overlay) and TFEB knockdown (blue overlay) cell lines. A single representative histogram is shown, with mean fluorescence intensity ± SEM presented in graph to the right (n = 4) ***p < 0.0005, **p < 0.005, *p < 0.05. [file Image_5.tif]
